# Heart rate variability in conscious neonatal swine: spectral features and responses to short-term intermittent hypoxia

**DOI:** 10.1186/1472-6793-6-5

**Published:** 2006-06-16

**Authors:** Anthony L Sica, Ning Zhao

**Affiliations:** 1 Department of Physiology and Pharmacology, SUNY Downstate Medical Center, Brooklyn, New York 11203, USA

## Abstract

**Background:**

Spectral analysis of the cardiac time series has been used as a tool for assessing levels of parasympathetic and sympathetic modulation of the sinoatrial node. In the present investigation we evaluated daily changes in heart rate variability spectra in conscious neonatal piglets that were either neurally intact (n = 5) or had undergone right stellate ganglionectomy (n = 5). The partial stellectomized animals and their intact litter mates were exposed to four days of intermittent hypoxia, each day comprising nine episodes of hypoxia alternating with nine episodes of normoxia. A time control group (n = 7) comprised animals from different litters that were not exposed to intermittent hypoxia. We hypothesized that exposure to intermittent hypoxia would increase sympathetic efferent neuronal modulation of heart rate variability spectra in neurally intact animals and in those with right stellate ganglionectomy, and that his effect would be observed in heart rate variability spectra computed from baseline recordings.

**Results:**

Overall, heart rate variability spectra during baseline conditions were dominated by high frequency activity, a reflection of parasympathetic efferent neuronal innervation and linkage to the ventilatory cycle manifested as respiratory sinus arrhythmia. Exposure to intermittent hypoxia did not alter daily baseline spectral features that would indicate an increase of sympathetic cardiac activity: low frequency (0.05 – 0.15 Hz) activity was unaffected and the ratio of low- to -high frequency activity remained less than unity indicating a predominance of high frequency activity. The resultant spectra were remarkably similar despite differences in cardiac sympathetic efferent neuronal innervation and experimental treatment. When spectra were computed from cardiac time series during representative hypoxic episodes, significant increases in activity across the low frequency region (0.05 – 0.15 Hz) of heart rate variability spectra were noted and were comparable in neurally intact animals and in those with right stellate ganglionectomy.

**Conclusion:**

The findings of this investigation provided important information regarding sympathetic efferent neuronal innervation of the heart during the neonatal period. Both neurally intact animals and those with right stellate ganglionectomy had equivalent increases of activity in the low frequency region of heart rate variability spectra during hypoxic stimulation. Such a finding demonstrated the capability of residual cardiac sympathetic neuronal innervation to affect functionally appropriate changes in cardiac chronotropy.

## Background

Variability is an inherent feature of the cardiac interval time series reflecting joint modulation of sinoatrial pacemaker activity by sympathetic and parasympathetic systems. Autonomic influences on heart rate variability (HRV) are readily apparent in autopower spectra computed from short records (3 – 5 min) of electrocardiographic R wave to R wave (R-R) intervals. Such spectra typically exhibited peaks in two distinct regions, one a low frequency (LF) region, located between 0.05 and 0.15 Hz, the other a high frequency (HF) region located at the breathing rate and associated with changes in R-R interval durations during the ventilatory cycle, i.e., the respiratory sinus arrhythmia (RSA) [16,29]. While LF activity is considered to represent sympathetic efferent cardiac innervation and HF activity due to parasympathetic efferent cardiac innervation, this functional separation is not totally supported by experimental evidence that suggested joint modulation of either region [1,11,16,32]. Even though interactions between sympathetic and parasympathetic systems can occur, conditions of imposed stress may alter one or the other of the spectral regions, indicating selective activation of the sympathetic or parasympathetic system. For example, stimulation of peripheral chemoreceptors by hypoxia augmented activity in the LF region [20,35,37], whereas activation of central chemoreceptors by hypercapnia augmented HF activity [36,37]. Hypoxia-induced elevation of sympathetic efferent neuronal activity is of interest because it may play an etiological role in the genesis of human cardiovascular disease [6,26].

Animal models of adult human disease in which hypoxia is thought to play a significant role, e.g., the obstructive sleep apnea syndrome, have relied upon long-term exposures to chronic-intermittent hypoxia in order to reproduce the hypertension associated with untreated obstructive sleep apnea [6,26]. However, lengthy stimulus exposures are unsuitable for cardiovascular studies in large developing mammals because they would be separated from their mothers during the exposure periods, thereby disrupting established feeding and sleeping schedules. Further, prolonged periods of stimulation may not be required to produce changes in physiological function. For example, several recent studies in neonatal swine showed that exposure to a short protocol (48 min/d for 4 d) of intermittent hypercapnic hypoxia was sufficient to evoke cell death in brainstem regions involved in cardiorespiratory regulation [14,15]. These findings raised the possibility that changes in neuronal circuits of neonatal piglets underlying cardiorespiratory control could occur following brief exposures to intermittent hypoxia (IH). Hence, one objective of the present study was to determine whether or not exposure to short-term IH would increase LF activity in HRV spectra, and consequently increase LF/HF ratios, the latter an index of the relative contributions of sympathetic and parasympathetic modulation to HRV [16]. A second objective was evaluate the extent to which right stellate ganglionectomy would alter LF activity and LF/HF values in HRV spectra. The latter objective will be pursued because anatomical studies in swine showed that sympathetic efferent innervation of the sinoatrial node originated from peripheral ganglionic sites, i.e., middle cervical and stellate ganglia bilaterally [10]. We hypothesized that baseline HRV spectra would exhibit increased activity in LF region and increased values of LF/HF ratios, reflecting exposure to IH on the previous day or days, and that such an effect would occur in both intact and right stellate ganglionectomized animals. A change in HRV spectra of such ganglionectomized animals, indistinguishable from sham operated litter mate controls, would demonstrate hypoxia-induced, functionally equivalent activity in anatomically distinct sympathetic efferent cardiac nerves.

Experiments were carried out using awake pigs since this species has been shown by us and others to be suitable for long-term monitoring of cardiovascular activity [11,12,17,33,38]. Further, the use of an awake animal model avoided anesthetic related effects on HRV; for example, anesthetized rats exhibited an inverse RSA wherein R-R intervals during inspiration had longer durations than those of expiration [4,19,34]. Piglets exposed to IH were either those with right stellate ganglionectomy (RSGX), or sham (SHAM) operated litter mate controls. RSGX was selected because animals with this type of denervation are distinguished from control animals by their characteristic change in cardiac chronotropy, Q-wave to T-wave (Q-T) interval prolongation [33,38]. A third group comprised animals from different litters who did not receive IH stimulation. This control (CON) group was used to evaluate changes in HRV spectra due to passage of time. R-R interval time series of CON animals were also used to determine the relationships between interval durations and the ventilatory cycle because CON animals were likely to exhibit a more stable cardiac time series than those exposed to hypoxic stimulation.

## Results

**Figure 1:**
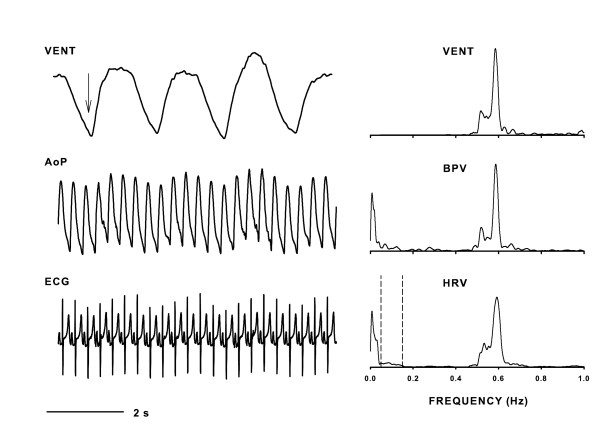
Left panel shows digital traces of ventilation (VENT), abdominal aortic blood pressure (AoP), and Lead II electrocardiogram (ECG) signals representing activity during baseline condition. Downward pointing arrow indicates inspiration. Right panel illustrates VENT spectrum along with the blood pressure variability (BPV) and heart rate variability (HRV) spectra (bin widths = 0.005 Hz). These spectra were computed from 150 s of continuous data that was free of arrhythmias and movement artifacts. The dashed lines in the HRV spectrum encompass the frequency boundaries for low frequency activity, i.e., 0.05 – 0.15 Hz. Note that AoP and HRV spectra exhibited ventilatory related activity that greatly exceeded the magnitudes of low frequency activity.

Telemetry recordings (examples from a CON animal in Fig. 1, left panel) were obtained from 17 animals, 5 animals each in the SHAM and RSGX treatment groups, and 7 animals in the CON group. Ventilatory frequency during baseline recordings did not differ significantly between treatment groups and ranged from 30 to 58 breaths per minute, with RSGX animals exhibiting a tendency to breathe more rapidly than other animals. As expected, RSGX animals exhibited significantly longer durations of R-R, Q-T, and corrected Q-T (Q-Tc) intervals than those of SHAM or CON animals (see Table 1).

**Table 1:** Group median durations (seconds) of Q-T, R-R, and corrected Q-T (Q-Tc) intervals measured in animals with right stellate ganglionectomy (RSGX), sham (SHAM) operated control animals, and time control (CON) animals.

**TREATMENT GROUP**	**BASELINE**			**HYPOXIA**		
	**R-R**	**Q-T**	**Q-Tc**	**R-R**	**Q-T**	**Q-Tc**
RSGX	0.342*	0.191*	0.330*	0.288†	0.168†	0.313
SHAM	0.294	0.171	0.313	0.267†	0.160†	0.309
CON	0.317	0.171	0.304			

*Interval durations of RSGX animals at baseline were significantly longer than those of either SHAM or CON groups.

†Within groups comparisons of interval durations, baseline vs. hypoxia, showed significant decreases in durations during hypoxia in RSGX and SHAM animals.

Statistical evaluation of HRV spectral features, areas of LF or HF regions, and LF/HF ratios, showed no effect of daily exposures to IH in either RSGX or SHAM treatment groups, neither was there any effect of passage of time on HRV spectra of CON animals. Hence, all statistical comparisons described below were made using data accumulated over the four baseline days

### Baseline HRV spectra

**Figure 2:**
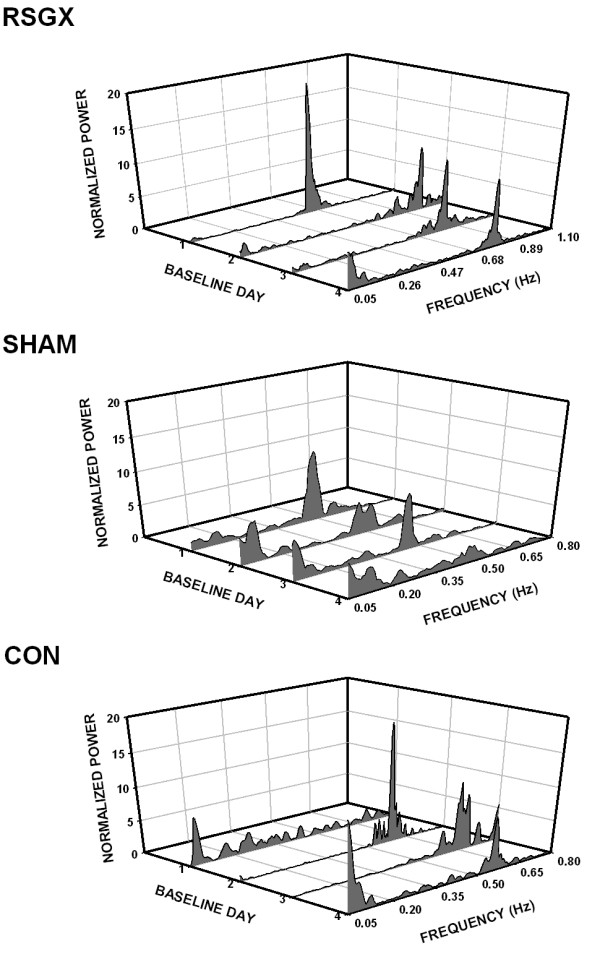
Three dimensional Waterfall plots of heart rate variability (HRV) spectra (bin width = 0.005 Hz) illustrate the relative magnitudes of low and high frequency activities on each of four baseline days. Each plot depicts HRV of a representative animal in each treatment group: animals with right stellate ganglionectomy (RSGX) exposed to intermittent hypoxia (IH), sham (SHAM) operated control animals exposed to IH, and time control (CON) animals with no IH exposure. In all plots low frequency activity occurred in the 0.05 – 0.15 Hz band, whereas the locations of high frequency activity were variable depending upon the ventilatory rate. For example, high frequency peaks were located at frequencies > 0.6 Hz for the RSGX animal, > 0.35 Hz for the SHAM animal, and > 0.5 Hz for the CON animal. Note there was an absence of high frequency activity on baseline day one in the CON animal.

**Figure 3:**
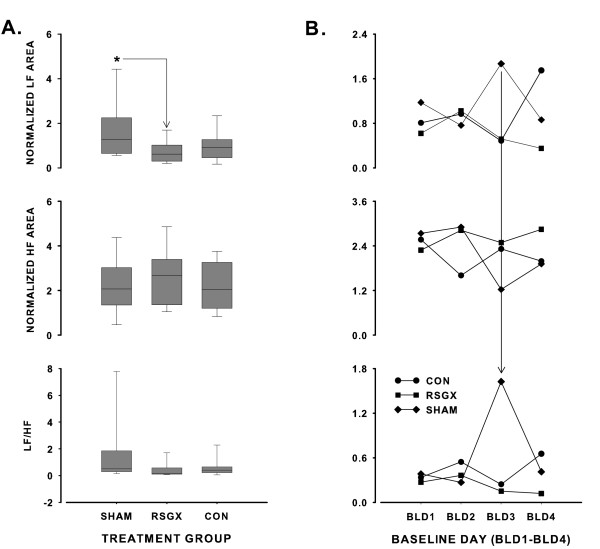
Panel A depicts vertical Box and Whisker plots of normalized low frequency (LF) power, normalized high frequency (HF) power, and resultant LF/HF ratios for the right stellate ganglionectomy group (RSGX), sham (SHAM) operated control group, and the time control (CON) group. Each box contains a horizontal line indicating the median value of the treatment group, the lower and upper boundaries of each box represent the 25^th ^and 75^th ^percentile values, and the lower and upper whiskers (resembling error bars) depict the 5^th ^and 95^th ^percentiles. The asterisk in the top graph indicates that LF power of SHAM animals differed significantly from that of RSGX animals. Panel B shows LF power (top), HF power (middle), and LF/HF ratio (bottom) on each baseline day (BLD1–BLD4) for each treatment group. The vertical solid line extending from top graph to bottom graph points to the marked increase in LF/HF ratio on BLD3 for the SHAM group.

Examination of HRV spectral features showed many similarities despite differences in treatment groups. Frequency locations of LF and HF peaks in HRV spectra were similar in each treatment group (see Table 2). Examination of representative HRV spectra showed that HF activity was generally greater in magnitude than LF activity (Fig. 1, right panel; Fig. 2). The area of HRV spectra encompassed by HF activity was usually larger than that of LF activity regardless of treatment group (*ca*. top and middle graphs of Fig. 3A). The predominance of HF activity in HRV spectra was quantified by comparisons of LF/HF ratios in each treatment group, median values computed over four baseline days were less than unity in all treatment groups, with RSGX animals exhibiting the lowest values (Figs. 3A and 3B, bottom graphs). A marked departure from the general trend of HF dominance was observed on baseline day three for SHAM group animals when a LF/HF ratio greater than unity was noted (Fig. 3B, bottom graph). This finding was due to a marked increase in LF area accompanied by a decrease of HF area of similar magnitude (Fig. 3B, top and middle graphs). This one time occurrence was sufficiently large to account for the finding of significance between RSGX and SHAM animals (Fig. 3A, top graph). Nevertheless, the latter result cannot be attributed to IH exposure because neither RSGX nor SHAM animals had either LF or HF areas that differed statistically from those of CON group animals (Fig. 3A and 3B, middle graphs).

**Table 2:** Locations of low and high frequency peaks (LF, HF; respectively) in baseline records of heart rate variability spectra in animals with right stellate ganglionectomy (RSGX), sham (SHAM) operated control animals, and time control (CON) animals. Data are given as the group medians, 25^th ^and 75^th ^percentiles.

**TREATMENT GROUP**	**LF PEAK (Hz)**	**HF PEAK (Hz)**
	**Median; 25%, 75%**	**Median; 25%, 75%**
RSGX	0.08; 0.06, 0.09	0.66; 0.5, 0.96
SHAM	0.08; 0.06, 0.10	0.52; 0.48, 0.67
CON	0.09; 0.07, 0.11	0.59; 0.56, 0.68

### Ventilation-related changes in R-R interval durations

**Figure 4:**
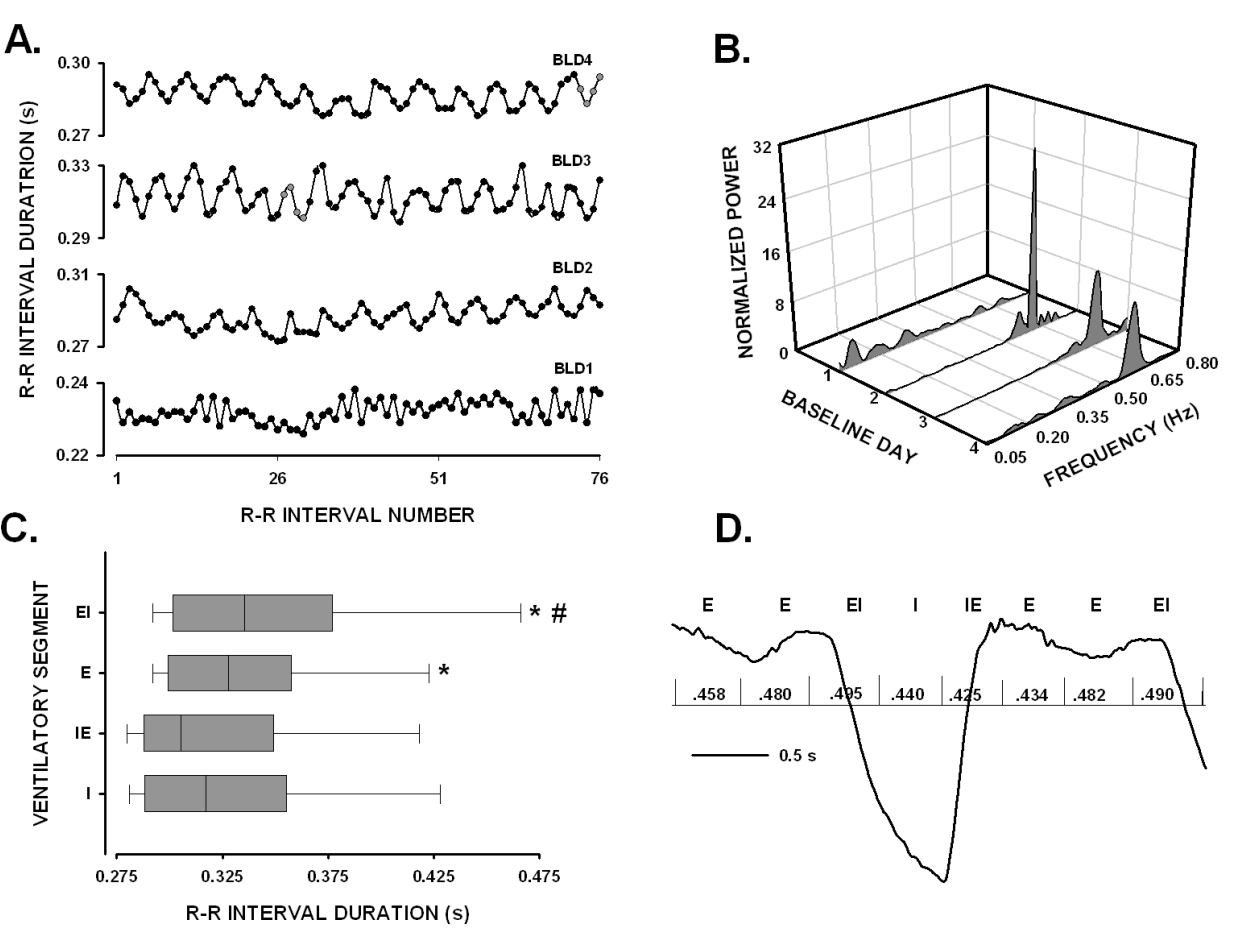
Panel A depicts R-R interval durations for an animal in the CON group over four baseline days (BLD1–BLD4). Panel B shows HRV spectra (bin width = 0.005 Hz) corresponding to full records (150 s long) of time series in Panel A. Panel C contains horizontal Box and Whisker plots of R-R interval durations for different segments of the ventilatory cycle: I, inspiration; IE, inspiratory to expiratory transition; E, expiration; EI, expiratory to inspiratory transition. The vertical line in each box represents the median value of R-R interval duration for each segment, the left and right boundaries of each box indicate the 25^th ^and 75^th ^percentile values, and left and right whiskers (resembling error bars) mark the 5^th ^and 95^th ^percentile values. Asterisks indicated that R-R interval durations occurring in either E or EI segments were significantly longer than those of either I or IE segments. The finding that R-R interval durations of EI segments were significantly longer than those of E segments is signified by the pound sign (#). Panel D shows a representative breath, inspiration down, with superimposed vertical lines indicating the temporal locations of R waves with reference to ventilatory segments: E, EI, I, and IE. The elapsed time between contiguous R waves, i.e., R-R interval durations, is given in seconds.

The observation that HF peaks in HRV spectra were present at the same frequency locations as those in VENT spectra was taken as an indication of ventilatory modulation of cardiac R-R intervals. Nonetheless, the finding that peaks in HRV and VENT spectra occupy the same frequency locations does not yield information regarding the phasic influences of ventilation on R-R interval durations. The temporal dispersion of R-R intervals across the ventilatory cycle was examined in seven CON animals that had VENT signals with well-defined boundaries between inspiratory and expiratory portions of the ventilatory cycle (examples in Fig. 1, left panel; Fig. 4D). Figure 4A shows a time series of R-R intervals for one animal on each of four successive baseline days (BLD 1–4), and demonstrates marked fluctuations in R-R interval durations on all baseline days except day one (BLD1). The oscillatory patterns represented shortening and lengthening of R-R interval durations during inspiratory and expiratory segments, respectively (see Fig. 4D). The autopower spectra of Figure 4B showed that HF activity dominated spectra on days two through four when changes in R-R interval durations had marked oscillatory patterns (Fig. 4A), whereas only low frequency activity occurred on baseline day one when R-R interval durations exhibited little variation. R-R interval durations were evaluated by selecting recordings on one baseline day and then sorting R-R interval durations according to time of occurrence in the ventilatory cycle as depicted in Figure 4D. Figure 4C shows median R-R interval durations in each ventilatory segment. R-R intervals that occurred during expiratory phases or during the expiratory to inspiratory transitions had significantly longer median durations (0.328 and 0.336 s, respectively) than those of either inspiratory phases or inspiratory to expiratory transitions (0.317 and 0.305 s, respectively). Also, durations of R-R intervals in the expiratory to inspiratory transition were significantly longer than those of expiration; however, no such difference was noted between inspiratory R-R intervals and intervals in the inspiratory to expiratory transition.

### Changes in cardiac intervals and HRV spectra during IH

**Figure 5:**
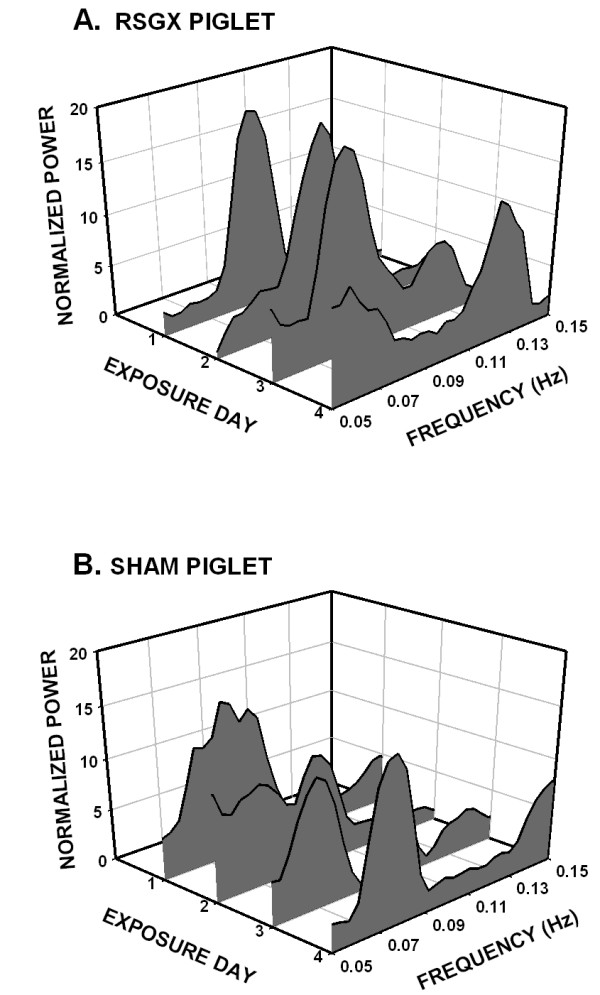
Heart rate variability spectra (bin width = 0.005 Hz), on four exposure days, were truncated to place focus on activity in the low frequency range, 0.05 – 0.15 Hz of a representative RSGX (A.) and SHAM (B.) animal. Spectra were constructed from 150 s long records taken during the final episode of hypoxia on each exposure day. Note that normalized power is scaled identically for each spectrum and that levels of activity were equivalent for each animal.

RSGX and SHAM animals had similar changes in cardiac chronotropy during hypoxia. Both groups of animals exhibited decreases in R-R and uncorrected Q-T interval durations during hypoxia that differed significantly from their respective baseline values (see Table 1). However, the magnitudes of such changes were similar within each group, yielding nearly identical Q-Tc interval durations (Table 1). Likewise, comparable changes were also noted in HRV spectra that were computed during either the 8^th ^or 9^th ^episode of hypoxia on each baseline day for each RSGX and SHAM animal. The HRV spectra of Figure 5 illustrate activity in the LF region of a RSGX animal and a SHAM animal during a representative hypoxic episode on each exposure day. It is noteworthy that levels of LF activity during hypoxia were comparable in HRV spectra of RSGX and SHAM animals. While both groups of animals showed significant increases of LF activity during hypoxia compared to baseline activity, the relative change in activity from baseline was similar in each group (see Table 3). A comparison of HF activity during baseline and hypoxia was not possible since activity in the HF region was usually not observed due to the relatively high ventilatory rates (96 – 156 breaths per minute) observed during hypoxic episodes.

**Table 3:** Spectral area encompassed by low frequency activity (0.05 – 0.15 Hz) for right stellectomized (RSGX) and sham operated controls (SHAM) during baseline and hypoxia, 8^th ^or 9^th ^episode of intermittent hypoxia. Data are given as the group medians, 25^th ^and 75^th ^percentiles.

**CONDITION**	**RSGX**	**SHAM**
	**Median; 25%, 75%**	**Median; 25%, 75%**
BASELINE	0.5; 0.21, 0.94	1.3; 0.74, 2.24
HYPOXIA	1.8; 1.01, 2.25*	2.8; 1.78, 3.74*
% INCREASE: BASELINE → HYPOXIA	285.0; 62.88, 617.19	76.5; -0.42, 338.7

*Low frequency area during hypoxia was significantly larger than that of baseline, i.e, normoxia.

## Discussion

We hypothesized that IH would evoke changes in cardiac beat-to-beat dynamics of neonatal swine such that indices of sympathetic activation, increased LF areas and LF/HF ratios greater than unity, would emerge over the course of the IH protocol. Intermittent hypoxia did not alter any index of HRV spectral activity whether piglets were intact, i.e., SHAM group, or had undergone RSGX. In fact our finding that LF areas of SHAM animals were significantly larger than those of RSGX animals was found to be a result of increased LF activity on a single day; despite such an occurrence LF areas of SHAM animals were not different from those of CON animals who breathed room air. Such similarity in regulation of cardiac chronotropy was also apparent during hypoxic episodes. During IH, RSGX and SHAM animals had similar increases in LF activity of HRV spectra and similar durations of Q-Tc intervals. These findings supported the hypothesis that a stressor recruited sympathetic efferent neuronal activity from contralateral intrathoracic neurons to affect the sinoatrial node [10]. Additionally, this residual sympathetic innervation could have altered activity of postganglionic parasympathetic neurons that innervated the sinoatrial node as demonstrated in in vitro studies of right atrial ganglionic neurons in neonatal swine [28].

The observation that conscious neonatal piglets exhibited changes in R-R interval durations during baseline conditions, indicative of RSA, *is *novel, but not unexpected as RSA is a prominent feature of R-R interval time series in a number of different mammalian species including human infants and adults (citations in references [4,18]). Such a pervasive feature suggests a common neuroanatomical organization [31]. In fact, we and others have shown that the organization of the brainstem vagal motor complex in piglets was similar to that of other species, with cardioinhibitory neurons located in the nucleus ambiguus [10,21]. Electrophysiological studies in felines showed that cardioinhibitory neurons in the nucleus ambiguus exhibited inhibitory postsynaptic potentials during inspiration followed by depolarization and action potentials during early and late expiration [8]. Likewise, whole cell recordings of nucleus ambiguus neurons in an en bloc neonatal rat brainstem preparation revealed an inspiration-related inhibition that was associated with marked increases of gabaergic and glycinergic inhibitory postsynaptic currents [18]. The aforementioned studies provided strong support for a neural origin for RSA that involved phasic interactions between brainstem neurons involved in shaping central respiratory activity and those providing parasympathetic efferent preganglionic outflows to postganglionic neurons innervating sinoatrial tissue. Hence, the progressive increase in R-R interval durations of piglets during the time course of expiration (depicted in Figs. 4C and 4D) may be viewed as representative of the expiration-related depolarization exhibited by feline nucleus ambiguus neurons [8]. Our finding that R-R intervals with the longest durations occurred at the expiratory to inspiratory transition and those with the shortest durations at the inspiratory to expiratory transition may simply represent species and/or maturational differences in durations and intensities of inhibitory and depolarizing currents. It is also likely that age-related differences in organization of the respiratory phases may have influenced the durations of R-R intervals. For example, we did not observe phrenic activity in earlier studies of neonatal cats or piglets [24,25] that corresponded to post-inspiration phrenic discharge (i.e. Stage 1 expiration) during which cardioinhibitory neurons in mature cats fired action potentials [8] An absence of post-inspiratory activity could mean that the temporal course of inspiration-related inhibition in the neonatal piglet is different, and this difference could explain the temporal dispersion of R-R intervals across the respiratory cycle. The absence of post-inspiratory activity suggested that inspiration-related inhibition included the transition zone between inspiratory and expiratory phases. In fact, our finding that R-R interval durations were shortest during this transition supported that idea. A change in the timing of phasic inhibition would be accompanied by changes in the temporal features of expiration-related depolarization such that it might include the beginning of the inspiratory phase, thereby, accounting for the occurrences of R-R intervals with the longest durations at the expiratory to inspiratory phase transitions. Further such an overlap of expiration-related depolarization with the onset of inspiration would delay the appearance of phasic inhibition of cardioinhibitory neurons as indicated by the observation that significant shortening in durations of R-R intervals occurred first during mid-inspiration (see Fig. 4D). The foregoing neural schema, while speculative, provides a reasonable explanation for the respiratory-related differences in R-R interval durations noted in this study.

A shortcoming of our investigation was that we did not fully evaluate the sources of RSA by application of ganglionic blockade. Nevertheless, substantial evidence for a neural origin for RSA was provided by animal and human studies that showed marked reduction of HF activity after parasympathetic blockade [1,11,17,39]. However, the possibility that mechanical effects of respiration, i.e., changes in intrathoracic pressure and/or cardiac filling, contributed to RSA cannot be discounted since RSA has been demonstrated in studies of transplanted human hearts, albeit at a much reduced level compared to normal control subjects [3,27,30].

## Conclusion

Our study provided important information regarding autonomic modulation of HRV during the first month of life for the piglet, a time frame generally accepted as equivalent to periods of vulnerability during the first six months of human life [5,13,23]. First, the equivalence of LF activity in SHAM and RSGX animals on baseline days and during episodes of intermittent hypoxia demonstrated that functionally appropriate regulation of cardiac chronotropy could be attained by recruitment of residual intrathoracic sympathetic ganglionic neurons. Second, the presence of RSA in the cardiac time series of developing mammals has important functional consequences. RSA has been hypothesized as a mechanism whereby changes in R-R interval durations occur at appropriate times in the ventilatory cycle so that ventilation to perfusion ratios are optimized, making for efficient exchange of pulmonary gases as demonstrated in adult humans and dogs [7,9,22]

## Methods

This research was approved by our Institutional Animal Care and Use Committee. The experimental procedures complied with the "Guiding Principles in the Use and Care of Animals" approved by the Council of American Physiological Society and also with Federal and State regulations. Near-term pregnant sows (*Sus scrofa*) were acquired from Parson's Farm in Massachusetts and delivered to our Division of Laboratory Animal Resources. Sows gave birth with no complications, and piglets were housed with their respective sows for the duration of each study, sows were then returned to Parson's farm.

### Surgical procedures

Piglets ranging in age from 7 – 16 d, and from 2.2 – 5.0 kg in weight underwent the various surgical procedures described below. Briefly, two to five days after birth, animals were anesthetized with Saffan (2 ml/kg, i.m.), a catheter was placed in a lateral ear vein for continuous infusion of 5% Dextrose – lactated Ringer's solution (6–11 ml/kg/h), and for periodic administration of Saffan (68 mg/kg/30 min). All piglets were intubated and ventilated mechanically with 100% O_2 _at tidal volumes of 10.0 – 15.0 ml/kg and at frequencies ranging from 24 – 40 strokes/min. The electrocardiogram (ECG) was monitored throughout each procedure. Core body temperature was measured and kept within normal limits (38-39°C) using a servo-regulated heating system. The details of our sterile surgical protocols are described in more detail in our previously published studies [33,38].

RSGX and SHAM operated groups (n = 5 animals per group). For animals undergoing RSGX, the thoracic wall was opened along the mid-sternal line and the entire sternum was divided. Following exposure of the right stellate ganglion, all nerves entering and exiting the ganglia were isolated, ligated, and cut. The ganglion was then removed and stored in 10% formalin for later histologic study. Animals serving as SHAM operated controls were litter mates of RSGX animals, and underwent similar surgical procedures, i.e., dissection to the level of the right stellate ganglion, but no manipulation of the ganglion or its innervation. The sternum, subcutaneous muscles, and skin were then sutured by layers.

Following thoracic surgery, telemetry transmitters (PhysioTel ^® ^Multiplus™, type TL11M3-D70-PCPT, Data Sciences International, St. Paul, MN) were placed into a subcutaneous pouch made on the lower part of the right lateral abdomen. Transmitter sensors for ECG recording were tunneled subcutaneously from the site of the pouch and placed in a Lead II configuration. Next, a pressure sensitive catheter was inserted through the right femoral artery and advanced into the abdominal aorta for recording aortic blood pressure (AoP). A second pressure sensitive catheter was then inserted subcutaneously through a small opening made on the right lateral thoracic wall to record changes in intrathoracic pressure, a measure of ventilation (VENT). The tips of each sensor were securely sutured to adjoining tissues, and all wounds closed with non-absorbable sutures.

CON group (n = 7 animals). This group comprised animals chosen from litters other than those used for selection of RSGX and SHAM animals. These animals did not undergo thoracic surgery, but did undergo surgery for implantation of telemetry transmitters.

Following the completion of surgical procedures, anesthesia was discontinued, piglets were extubated and carefully monitored until they were ambulatory. Antibiotics (Bicillin C-R, 0.2 ml/day; Amiglyde-V, 5 mg/kg/day) and an analgesic (Buprenex, 0.005 ml/kg/day) were administered immediately after completion of surgery and over the next three days. All medications were provided by our Division of Animal Laboratory Resources.

### Experimental protocol

Four to seven days postoperatively, a RSGX animal and a SHAM operated control animal were brought to our laboratory for exposure to IH. Animals were placed in adjoining Lucite chambers (12.5" w, 12" h, 19" l) fitted with diffusers to distribute gas, and with small vents to allow egress of gas to prevent build-up of pressure within boxes. Hypoxic exposures were made over four successive days, after a morning feeding, and carried out at room temperature. For technical reasons (see next section), simultaneous recordings of signals from animals in nearby chambers were impossible. Hence, one animal of each pair was randomly selected to receive hypoxia first. Prior to hypoxic exposures animals were allowed to adapt to the novel environmental conditions. Once the animals were well-adapted to the chamber, i.e., lying prone with eyes open, a 5 min baseline recording was made. Chamber oxygen and carbon dioxide content were monitored continuously by an oxygen sensor (KE-25, Kent Scientific Inc.), and a carbon dioxide monitor (Ohmeda 5200). The IH protocol comprised 9 episodes of hypoxia alternating with 9 episodes of normoxia, each episode lasted 4 min. This pattern of alternating gas exposures was controlled by hardware and software developed in our laboratory. During hypoxia, chamber oxygen concentration was lowered rapidly from 20 – 21% O_2 _to 6 – 7% O_2_, and then held at that level for three minutes, after which chamber oxygen concentration was rapidly increased to 20 – 21% O_2_, and maintained at that level for three minutes. Transitions from room air to hypoxia and from hypoxia to room air required one minute. All signals were monitored continuously throughout each recording session and for at least 30 min after terminating the final hypoxic episode of each day.

Animals in the CON group were placed in exposure chambers on four successive days. Following adaptation and baseline recording, the temporal sequence of events was identical to those experienced by RSGX and SHAM animals, with the exception that transitions were from room air to room air. Hence, these animals experienced the same environmental stimuli as IH exposed animals, but no exposure to hypoxia.

Piglets were separated from their sows for approximately 2 h on days one through three of the four day protocol. On day four, approximately two hours after the termination of the experimental protocol, animals were anesthetized with Nembutal (70 mg/kg, i.p.) and heart, lungs, and kidneys were harvested for detection and amplification of neuronal nitric oxide synthase using a semi-quantitative PCR technique. These data will be reported in another manuscript.

### Data acquisition

Signals were transmitted over a non-commercial portion of the amplitude modulated radio band. As each transmitter uses the same broadcast frequency, monitoring animals in close proximity to one another would induce cross-talk between signals broadcast from individual transmitters. Hence, it was necessary to carry out the experimental protocol one animal at a time. Telemetry receivers (RMC-1, Data Sciences International, Inc.) were placed beneath each exposure chamber and were connected to a data exchange matrix (Data Sciences International, Inc.) that transmitted signals to the acquisition system via a local area network type cable. A continuous measure of laboratory barometric pressure was provided by an Ambient Pressure Reference monitor (APR-1, Data Sciences International, Inc.) and corrected pressures recorded by AoP and VENT pressure catheters. Telemetry signals were acquired at a rate of 1 kHz using commercially available hardware and software (Dataquest™ A.R.T., Data Sciences International, Inc.). Baseline recordings were reviewed for the presence of arrhythmia and/or excessive movement artifacts. Records containing such events were not analyzed further.

### Data analysis

All graphical and spectral analyses were carried out using DADiSP/2002™ (DSP Development Corp.) The effect of RSGX on cardiac chronotropy was demonstrated by computing the durations of Q-T and R-R intervals in five non-successive cardiac cycles on each baseline day. Non-contiguous intervals were used to any local trends in the data. Likewise, measurements of O-T intervals were made during the final episode of hypoxia on each exposure day. Q-T interval durations were measured graphically by displaying the digitized ECG file, and by manually placing software marks at the beginning of the Q-R-S complex and the end of the T wave. The duration of the uncorrected Q-T interval was computed as the time difference between the two software marks. Likewise, marks were place at the peaks of the appropriate R waves, and the temporal differences between adjacent R waves gave the R-R interval durations. These measurements were then used to obtain heart rate corrected Q-T interval durations using Bazett's [2] formula: Q-Tc = Q-T/(R-R)^½^.

To determine whether ECG R-R interval durations demonstrated respiratory modulation, the ventilatory cycle was partitioned into four segments: inspiration, transition from inspiration to expiration, expiration, transition from expiration to inspiration. This was accomplished by first using a level-crossing algorithm to position software generated pulses at the temporal locations of peaks in R waves in approximately 200 cardiac cycles. Next, R-wave derived pulses were plotted along with corresponding respiratory cycles, and R-R interval durations computed for each ventilatory segment.

Spectral analysis was carried on all signals by computing power spectral density histograms, bin widths were 0.005 Hz in all cases. Each recording was truncated to a record of continuous data, 150 s in duration, and free of arrhythmias and/or movement artifacts. Best power of two power spectral density histograms were then computed, and smoothed using a five point moving average. Last, each spectrum was normalized by dividing each bin by the total spectral area computed using Simpson's rule. In this manner, area under the curve was derived for the LF region, 0.05 – 0.15 Hz (28 bins), and for the HF region, 28 bins encompassing the ventilatory related peak. As well, LF/HF ratios were computed to evaluate the relative sympathetic – parasympathetic contribution to HRV.

Spectral analysis of the systolic portion of the AoP signal was performed by first positioning software generated pulses at the peaks of systole using the peak finding function in DADiSP, thereby creating a file of voltage values of peaks and the times of their occurrences. Similarly, pulses were positioned at the peaks of R waves, thereby creating a file of voltage values and times of occurrences. These data were demeaned, convolved with a Hanning window to reduce sidelobe leakage, and autopower spectra computed to give systolic AoP variability and HRV. Heart rate (HR) was given by the location of the primary peak in the systolic AoP autopower spectra. For the VENT signal, data were demeaned, convolved with a Hanning window, and then used to compute autopower spectra histograms. The locations of primary peaks in VENT spectra gave ventilatory frequencies.

### Statistical analysis

Statistical comparisons were made using nonparametric tests since most data were neither normally distributed nor were variances equivalent. Hence, data in tables are given as group median values along with 25^th ^and 75^th ^percentiles. Friedman's repeated ANOVA on ranks was used to evaluate changes in spectral features, e.g., area under the curve for LF and HF regions, and also LF/HF ratios that occurred over the four successive baseline days. Two group comparisons were made using the Mann-Whitney Rank Sum test and the Kruskal-Wallis ANOVA on ranks with post hoc Dunn's tests was used for three group comparisons. The Wilcoxon Signed Rank test for pairwise comparisons. Statistical tests were performed using SigmaStat v.3.1(Systat Software, Inc.) with level for statistical significance established at p < 0.05.

## Abbreviations

AoP – abdominal aortic blood pressure

CON – group of animals serving as time controls

ECG – electrocardiogram

HF – high frequency region of spectrum

HRV – heart rate variability

IH – intermittent hypoxia

LF – low frequency region of spectrum

LH/HF – ratio of low to high frequency area

Q-T – duration of interval from beginning of Q-wave to end of T-wave

QTc – corrected Q-T interval duration

R-R – duration of interval between adjacent R-waves

RSA – respiratory sinus arrhythmia

RSGX – group of animals with right stellate ganglionectomy

SHAM – control group comprising litter mates of RSGX animals

VENT – ventilation

## Authors' contributions

Author NZ performed all surgeries and was involved in data acquisition and database management. Author ALS oversaw and was involved in all experiments as well as in signal processing, statistical analysis, and writing the manuscript. Both authors read and approved the final version of this manuscript.
